# Supplementary material for: Machine Assisted Experimentation of Extrusion-Based Bioprinting Systems
Source: Micromachines (Basel). 2021 Jun 30;12(7):780. doi: 10.3390/mi12070780 (PMC8305959; doi:10.3390/mi12070780)
Supplement: Supplementary file 1 [file micromachines-12-00780-s001.zip › micromachines-1251063-supplementary.pdf]

Supplementary Materials: Machine Assisted Experimentation of Extrusion-based Bioprinting Systems

Shuyu Tian, Rory Stevens, Bridget T. McInnes, and Nastassja A. Lewinski

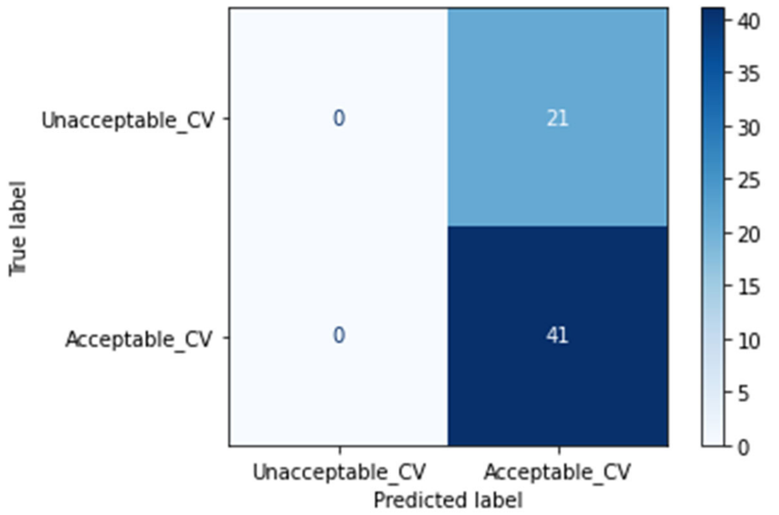

Figure S1. Confusion matrix of logistic regression and support vector classification of cell viability. 10% of the cell viability dataset was used as testing data while 90% of the dataset was used as training data. CV indicates cell viability.

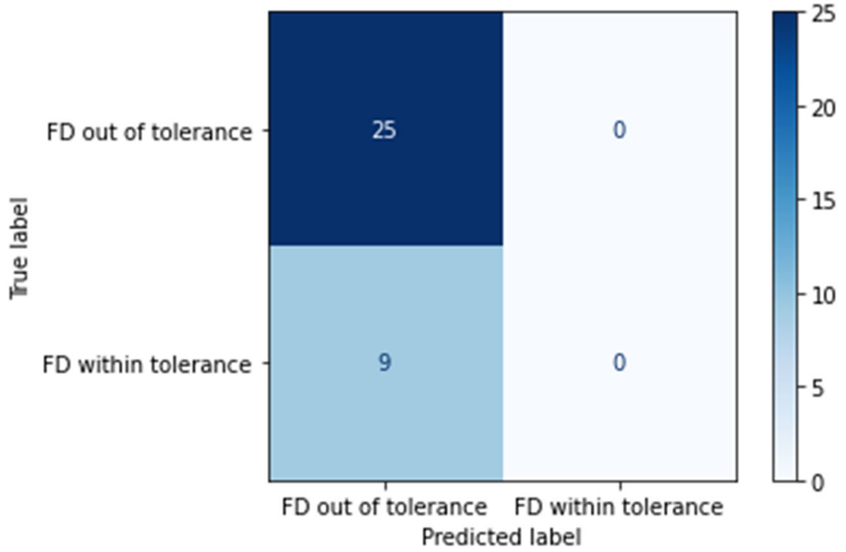

Figure S2. Confusion matrix of support vector classification of filament diameter. 10% of the cell viability dataset was used as testing data while 90% of the dataset was used as training data. FD indicates filament diameter.

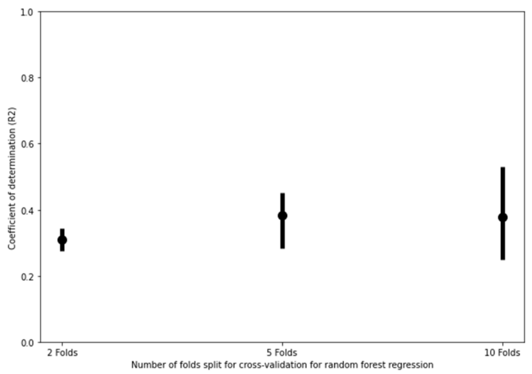

(**a**)

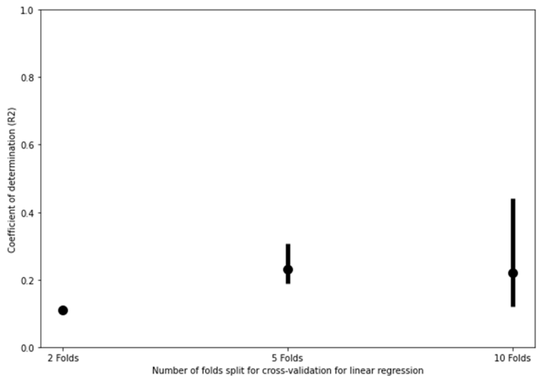

(**b**)

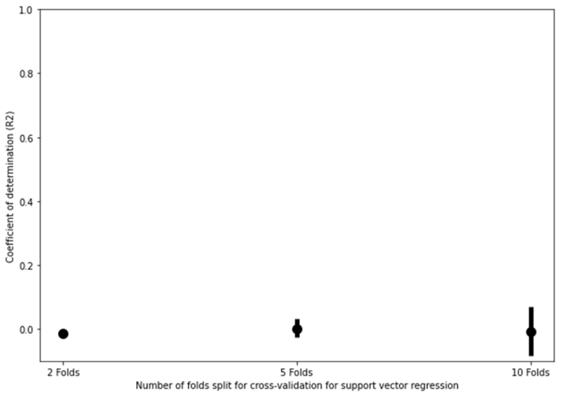

(**c**)

Figure S3. Coefficient of determination (R^2) values of cell viability regression models based on the number of folds tested for **b)** random forest regression, **b)** linear regression, and **c)** support vector regression. The upper and lower bounds of the error plots represent the maximum and minimum R^2 values produced for each fold division.

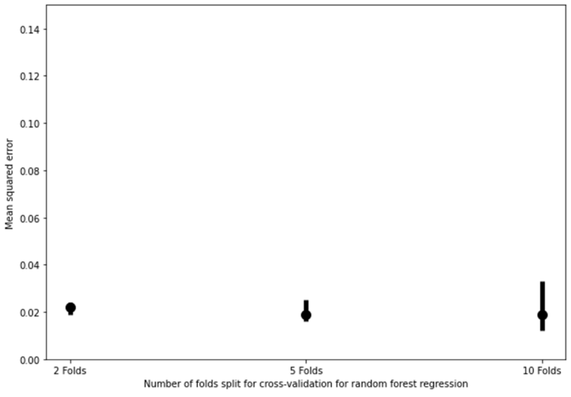

(**a**)

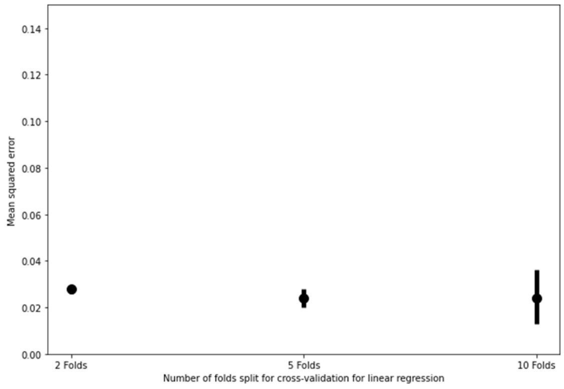

(**b**)

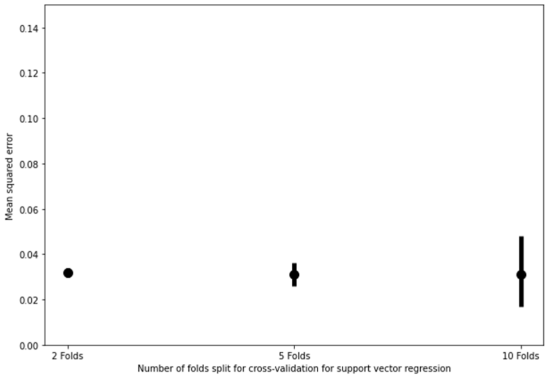

(**c**)

Figure S4. Mean squared error values of cell viability regression models based on the number of folds tested for **a)** random forest regression, **b)** linear regression, and **c)** support vector regression. The upper and lower bounds of the error plots represent the maximum and minimum mean square error values produced for each fold division.

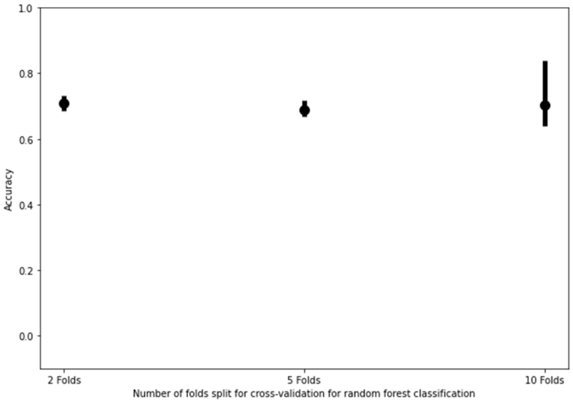

(**a**)

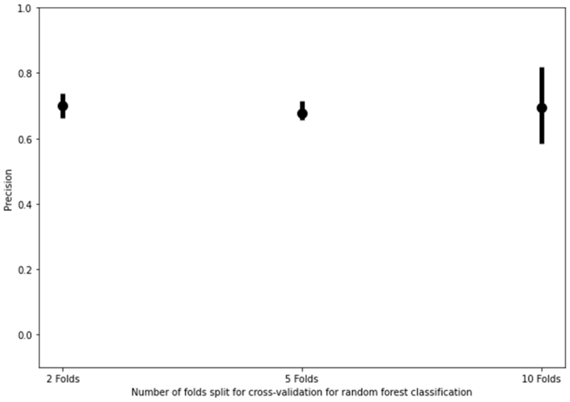

(**b**)

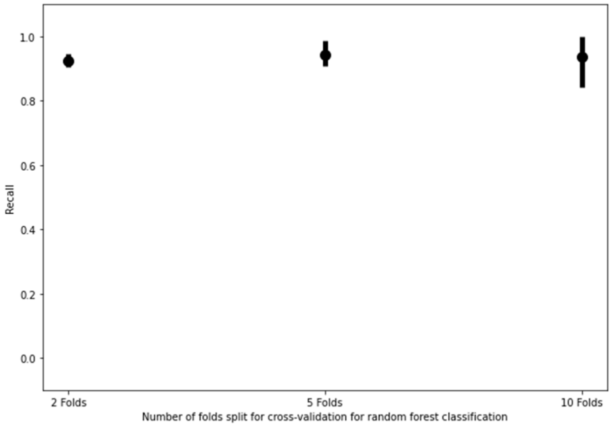

(**c**)

Figure S5. a) Accuracy, **b)** precision, and **c)** recall performance of the random forest classification cell viability model on different k-fold cross validation tests. The upper and lower bounds of the error plots represent the maximum and minimum metric values produced for each fold division.

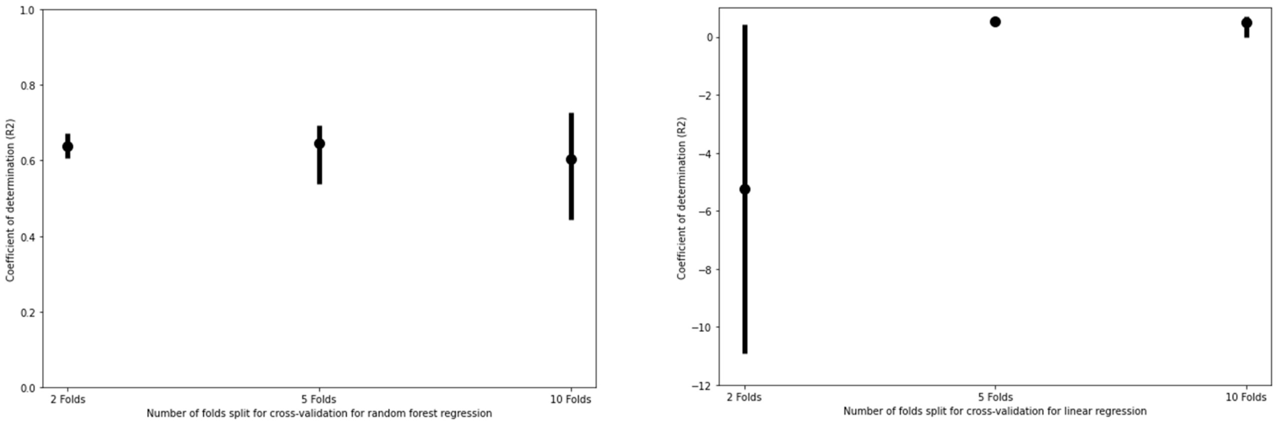

(**a**) (**b**)

Figure S6. Coefficient of determination (r^2) scores of filament diameter regression models based on the number of folds tested for **a)** random forest regression and **b)** linear regression. The upper and lower bounds of the error plots represent the maximum and minimum r^2 produced for each fold division.

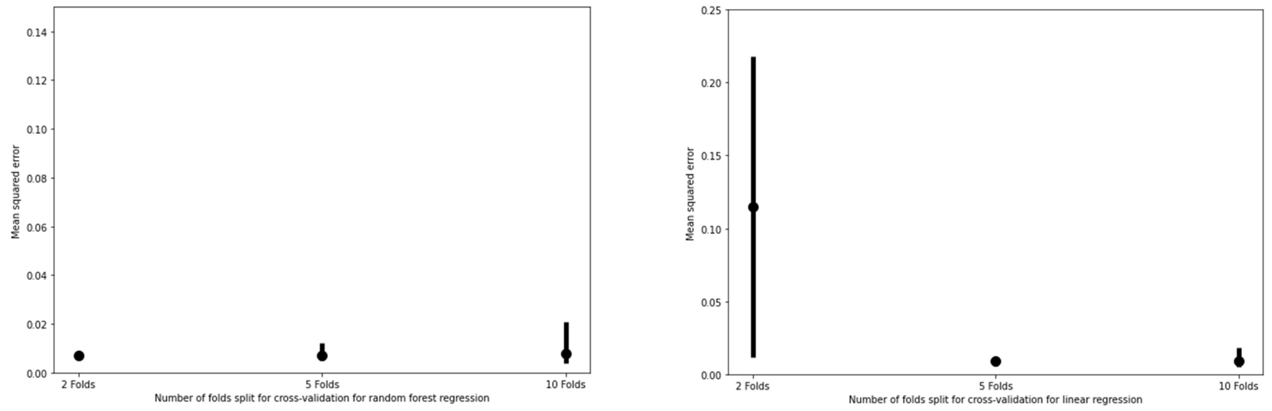

(**a**) (**b**)

Figure S7. Mean squared error scores of filament diameter regression models based on the number of folds tested for **a)** random forest regression and **b)** linear regression. The upper and lower bounds of the error plots represent the maximum and minimum mean squared error produced for each fold division.

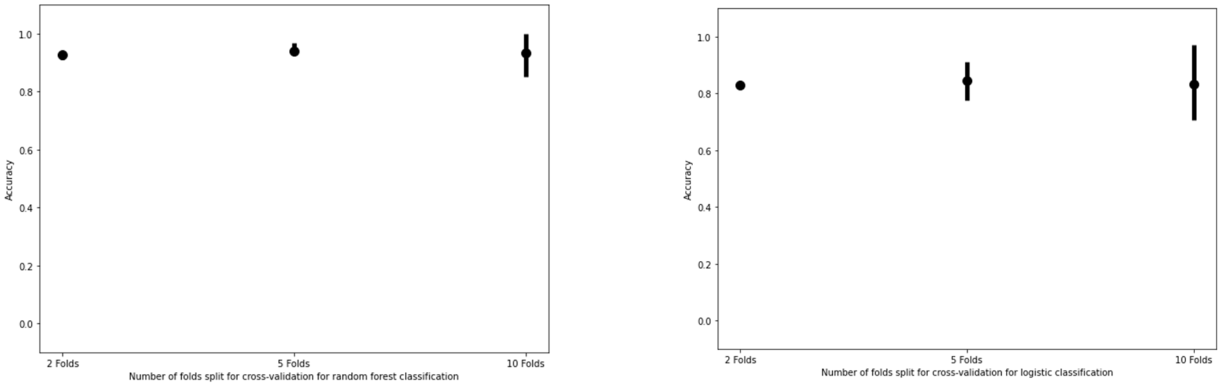

(**a**) (**b**)

Figure S8. Accuracy scores of filament diameter classification models based on the number of folds tested for **B)** random forest regression and **B)** logistic regression models. The upper and lower bounds of the error plots represent the maximum and minimum accuracy produced for each fold division.

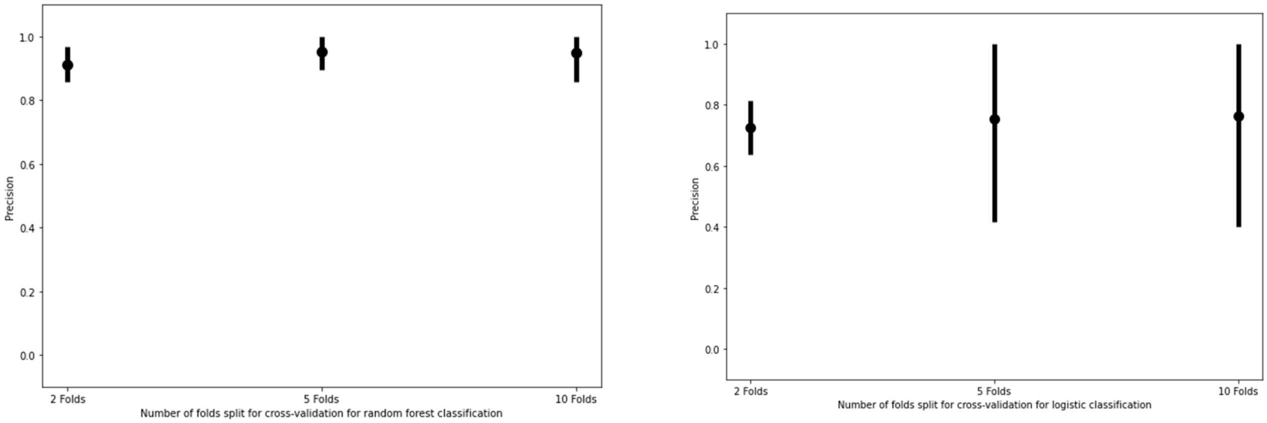

(**a**) (**b**)

Figure S9. Precision scores of filament diameter classification models based on the number of folds tested for **B)** random forest regression and **B)** logistic regression models. The upper and lower bounds of the error plots represent the maximum and minimum precision produced for each fold division.

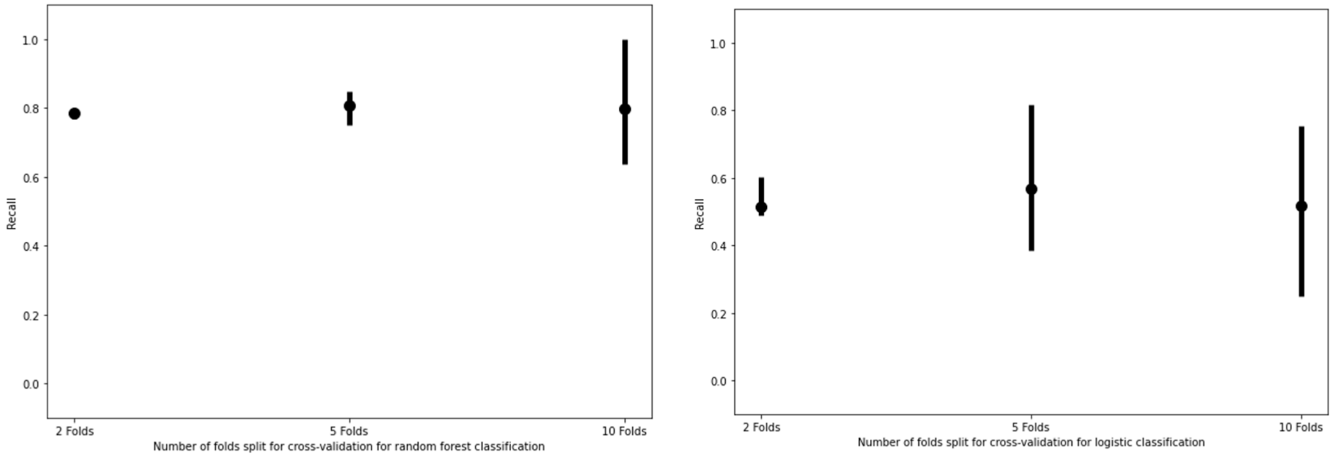

(**a**) (**b**)

Figure S10. Recall scores of filament diameter classification models based on the number of folds tested for **B)** random forest regression and **B)** logistic regression models. The upper and lower bounds of the error plots represent the maximum and minimum recall produced for each fold division.

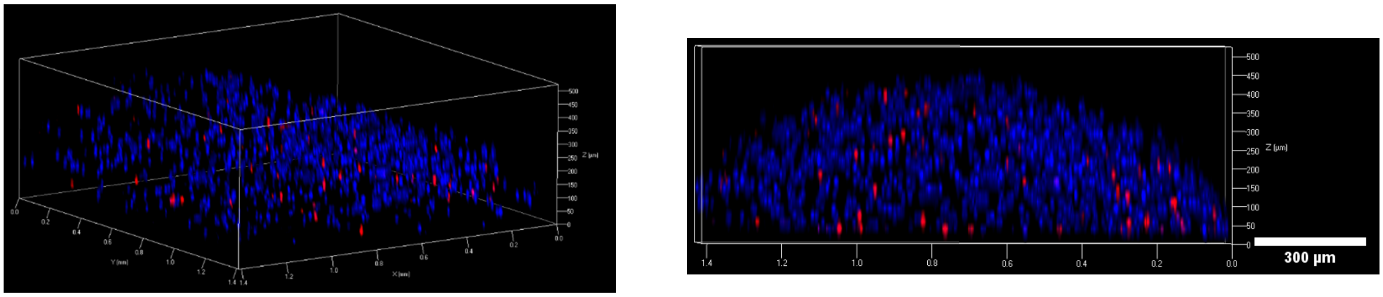

(**a**) (**b**)

Figure S11. Z-stack total/dead imaging of a portion of a filament from a 3/4 Alg/Gel printed construct (nozzle geometry = conical, nozzle diameter = 410 µm). An **A)** isometric view, **B)** cross-sectional view in the X-Z plane, and **C)** top view in the X-Y plane are shown at a magnification of 10x.

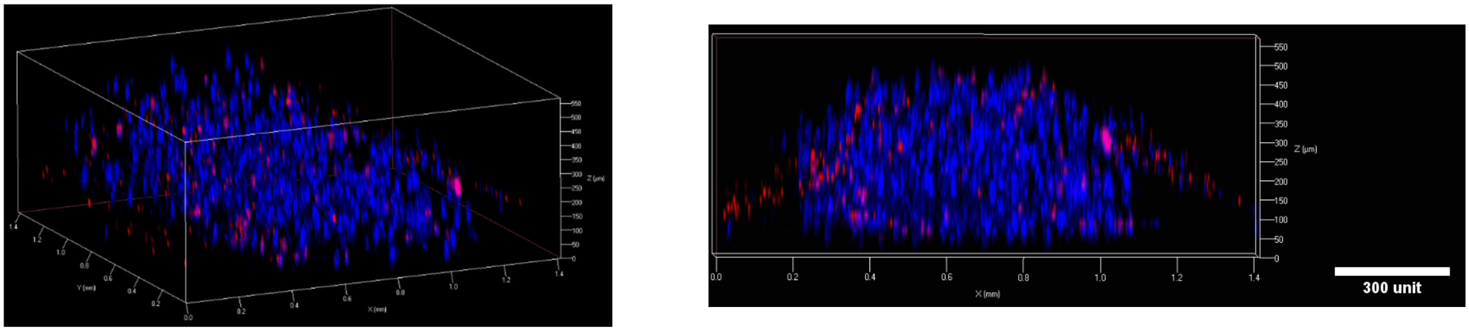

(**a**) (**b**)

Figure S12. Z-stack total/dead imaging of a portion of a filament from a 3/7 Alg/Gel printed construct (nozzle geometry = conical, nozzle diameter = 410 µm). An **A)** isometric view, **B)** cross-sectional view in the X-Z plane, and **C)** top view in the X-Y plane are shown at a magnification of 10x.

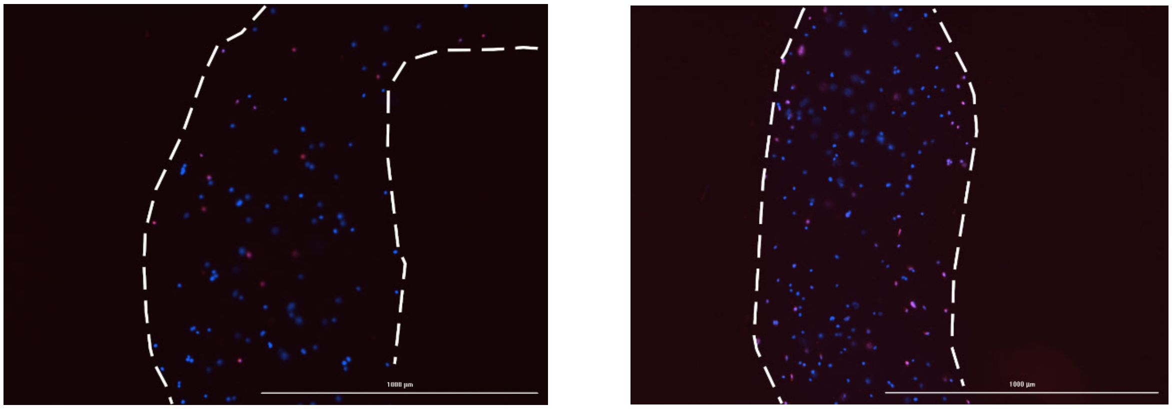

(**a**) (**b**)

Figure S13. Live/dead images taken on through the imaging plate reader immediately after extrusion of **a)** 3/4 Alg/Gel and **b)** 3/7 Alg/Gel. White borders indicate boundaries of the filament. The magnification of the images is at 4x.

Table S1. Predicted cell viability acceptability and actual cell viability acceptability comparison of 3/4 and 3/7 Alg/Gel constructs printed (nozzle geometry = conical, nozzle diameter = 410 μm).

Cell viability prediction model	Material concentration (%w/v)	Predicted cell viability acceptability (Yes/No)	Actual cell viability acceptability (Yes/No)
Random forest classification	3/4 Alg/Gel	Yes	Yes
	3/7 Alg/Gel	Yes	No
Logistic regression	3/4 Alg/Gel	Yes	Yes
	3/7 Alg/Gel	Yes	No
Support vector classification	3/4 Alg/Gel	Yes	Yes
	3/7 Alg/Gel	Yes	No

Table S2. Predicted tolerance and actual tolerance comparison of 3/4 and 3/7 Alg/Gel constructs printed (nozzle geometry = conical, nozzle diameter = 410 μm).

Filament diameter prediction model	Material concentration (%w/v)	Predicted tolerance condition	Percent error from nozzle diameter (410 μm) (%)	Actual tolerance condition
Random forest classification	3/4 Alg/Gel	Not within tolerance	126	Not within tolerance
	3/7 Alg/Gel	Not within tolerance	72.5	Not within tolerance
Logistic regression	3/4 Alg/Gel	Not within tolerance	126	Not within tolerance
	3/7 Alg/Gel	Not within tolerance	72.5	Not within tolerance
Support vector classification	3/4 Alg/Gel	Not within tolerance	126	Not within tolerance
	3/7 Alg/Gel	Not within tolerance	72.5	Not within tolerance

Table S3. Predicted extrusion pressure classifications compared against experimental outcomes for corresponding material concentrations of Alg/Gel. Actual values represent the mean ± standard deviation for all samples (n = number of batches).

Extrusion pressure prediction Model	Material and material concentration (%w/v)	Acceptable extrusion pressure predicted (Yes/No)	Actual extrusion pressure acceptability (Yes/No)
Random forest classification	3/4 Alg/Gel	Yes	Yes
	3/7 Alg/Gel	No	Yes
	8/20 Alg/Gel	No	No
Logistic regression	3/4 Alg/Gel	Yes	Yes
	3/7 Alg/Gel	Yes	Yes
	8/20 Alg/Gel	Yes	No
Support vector classification	3/4 Alg/Gel	Yes	Yes
	3/7 Alg/Gel	Yes	Yes
	8/20 Alg/Gel	Yes	No
